# ELEPHANTIASIS NOSTRAS VERRUCOSA: SUCCESSFUL SURGICAL TREATMENT IN A GROSS DEFORMITY

**DOI:** 10.4103/0019-5154.53178

**Published:** 2009

**Authors:** Purificación Gacto-Sánchez, P Fernandez-Ortega, JJ Pereyra-Rodríguez

**Affiliations:** *From the Departments of Plastic and Reconstructive Surgery, Virgen del Rocío University Hospitals, Sevilla, Spain*; 1 *From the Department of Dermatology, Virgen del Rocío University Hospitals, Sevilla, Spain. E-mail: purigacto@gmail.com*

Sir,

Elephantiasis nostras verrucosa (ENV) is a rare, chronic, deforming disorder characterized by hyperkeratosis and papillomatosis of the epidermis with underlying woody fibrosis of the dermis and subcutaneous tissue. No standard treatment for this rare cutaneous manifestation is available.[1] In this case report, we describe surgical treatment that is proved to be helpful when performed in addition to physiotherapy.

**Figure 1:**
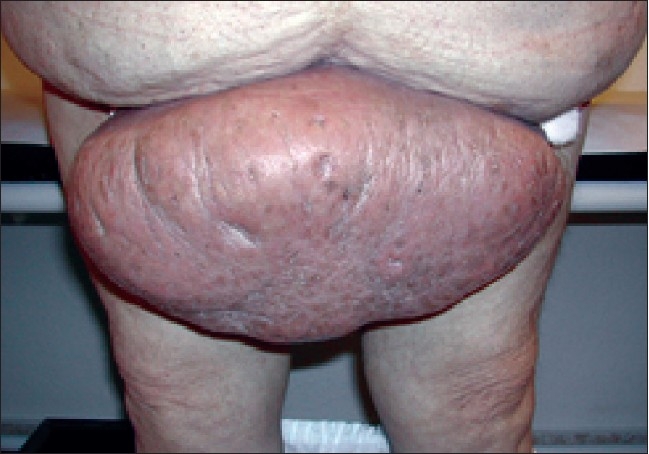
Pendulum-like mass arising from the anterior abdominal wall in its lower part covering penis and scrotal region

A 51-year old male was presented to our clinic with a 15-year history of a pendulum-like mass arising from the anterior abdominal wall in its lower part covering penis and scrotal region [Figure 1]. The developed gross deformity was causing significant psychological alterations as well as social isolation to the patient. His medical history showed significant hyperlipidemia, obesity, hypertension, and diabetes mellitus. There was no personal history of filariasis or Milroy's disease in his family.

**Figure 2:**
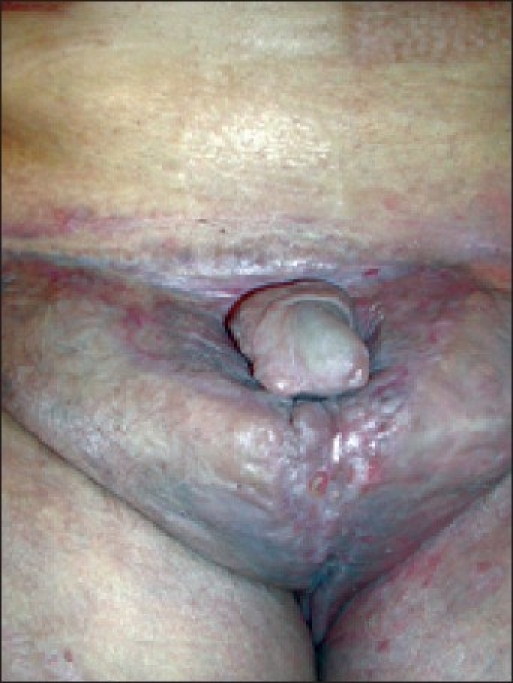
Penis and scrotal region 6 months after surgical treatment

Physical examination revealed generalized thickening and lichenification of the skin as well as multiple fistulas. No lymph nodes were palpable due to the woody nature of the tissue. Rectal examination revealed a slightly enlarged prostate without any palpable masses. Test results were negative for fecal occult blood. The patient underwent surgery to remove the mass. A compression stocking was used to treat the lymphedema. Ten months after the operation, we saw no signs of disease recurrence. The result was also aesthetically satisfactory [Figure 2].

Biopsy specimens revealed pseudoepitheliomatous hyperplasia with dilated lymphatic spaces in the dermis, accompanied by chronic inflammation and fibroblast proliferation. No evidence of neoplastic change was observed.

Elephantiasis is a clinical description of distinct cutaneous changes associated with a chronic underlying dysfunction in the lymphatic system. Many factors may lead to chronic lymphatic obstruction and stasis.[1] The common mechanism is an underlying lymphatic obstruction leading to impaired lymphatic drainage with abnormal accumulation of interstitial fluid and subsequent development of lymphedema. The overlying epidermis slowly develops a cobblestoned, verrucous appearance.[1,2] The precise role of some pathogenic organism, either bacterial or filarial, in “idiopathic” lymphatic obstruction is uncertain. Streptococcal and, less often, staphylococcal lymphangitis is considered to be the most likely causative agent.[1,2]

ENV and papillomatosis cutis carcinoides share many features. The latter can morphologically resemble ENV with single or multiple verrucous, partially ulcerated tumors on the chronically irritated edematous extremity. Both are histologically characterized by pseudoepitheliomatous hyperplasia. The diagnosis of papillomatosis cutis carcinoides is established by biopsy.[3]

Therapeutic efforts should aim to reduce lymph stasis, which will also improve the cutaneous changes. The mainstay of therapy remains prophylactic antibiotics, compression, and elevation of the affected limb.[3] Systemic retinoids, specifically etretinate, are useful in the treatment of diseases that are characterized by epidermal proliferation, as well as disorders of keratinization.[4] The value of topically applied tazarotene gel with its potent retinoid profile and low potential for systemic effects has still to be determined.[5] Our patient had a history of hyperlipidemia and refused systemic treatments. Hence, systemic retinoid therapy was deemed inappropriate.

ENV should be included in the differential diagnosis of chronic lymphedema and be differentiated from papillomatosis cutis carcinoides. Early diagnosis is paramount. Without appropriate early intervention, ENV continues to worsen and may result in gross deformity requiring amputation.[1,3]
